# Menstruation behaviour influencer model: a grounded theory of menstrual experiences of shame, embarrassment, stigma and absenteeism among pubescent girls in semi-urban and rural secondary schools in Enugu State, Nigeria

**DOI:** 10.11604/pamj.2023.45.47.39675

**Published:** 2023-05-18

**Authors:** Nneka Edith Ubochi, Ukamaka Anthonia Chinweuba, Njideka Peace Iheanacho, Easter Chukwudi Osuchukwu, Chijioke Oliver Nwodo, Anulika Jennifer Nnamani, Ngozi Phoeba Ogbonnaya, Vincent Nwokejiezi Ubochi

**Affiliations:** 1 Department of Nursing, Faculty of Health Sciences and Technology, College of Medicine, University of Nigeria Enugu Campus, Enugu State, Nigeria,; 2 Department of Nursing, University of Calabar, Cross Rivers State, Nigeria,; 3 Federal Neuropsychiatric Hospital, Enugu, Nigeria

**Keywords:** Grounded theory, menstruation, menstrual shame, stigma, pubescents

## Abstract

**Introduction:**

severally, studies had identified menstrual-associated shame, embarrassment, stigma, and absenteeism among pubescents in school with resultant challenges on their bio psycho-social functioning. However, what is not clear is the contribution of the home and school to the experiences. The objectives of the study were to explore the experiences with menstruation and menstrual hygiene management; explore the experiences with menstrual-associated shame, embarrassment, stigma, and absenteeism among participants; explore the bio-psycho-social issues associated with the experiences; understand the meaning of the experiences and propose a mid-range theory that explains the influences on pubescents´ menstrual behaviours.

**Methods:**

constructivist grounded theory design was used to explore the experiences of 20 purposively recruited pubescents from rural and semi-urban secondary schools. In-depth Interviews, focused group discussions, key informant interviews, and observations were employed to collect data until data saturation. Open and focused coding was conducted to identify emerging themes and sub-themes. These themes were returned to participants and literature for verification.

**Results:**

four (4) categories and eleven (11) sub-categories emerged from the data and formed four (4) themes that influence pubescents´ menstrual behaviour. They include: 1) individuals´ bio-physiological status, knowledge of menstrual health and menstrual characteristics; 2) regimenting school through strict rule enforcement, punishment/motivation, forced participation, and compliance; 3) scheduling academic activities/examination, sporting and other extra curricula activities; and 4) providing menstrual support by individual and institutional efforts to pubescents. Based on the relationship with other themes, the menstrual behaviour influencer model was proposed.

**Conclusion:**

menstrual influencers require the interaction between menstrual support and menstrual enablers by institutions for positive menstrual behavior. Failure to achieve this balance will lead to menstrual-associated shame, embarrassment, stigma, absenteeism, and school drop-out.

## Introduction

Menstrual-associated shame, embarrassment, stigma, and school absenteeism are well documented in the literature, however, the experiences of pubescents and the contributions of the home and school authorities to this menace among school-going pubescents are yet to be explored. Menstruation is a landmark in the process of growth and maturation and prepares adolescent girls for reproductive functions and motherhood [1], yet it is froth with biological, physical, social, and psychological challenges. The onset of mensuration (menarche) marks the period of biological and social transition from childhood to adulthood [2], hence menstruation and menstrual hygiene management (MHM) are issues that every adolescent girl of reproductive age faces in her life [3] irrespective of race, colour, place of residence or socio-cultural affiliation. Menstrual behaviours including shame, moodiness, excitement, embarrassment, pride, etc. exhibited by girls are associated with the cultural connotation of menstruation, the preparedness for menstruation, and the social support system available to her. Her resultant adaptive or maladaptive response could influence the confidence to speak out, negotiate health interventions; and or overcome the perceived feeling of distorted body image [4].

Adequate MHM is defined as “women and adolescent girls using clean menstrual management materials to absorb or collect blood that can be changed in privacy as often as necessary, for the duration of the menstruation period, using soap and water for washing the body as required and having access to facilities to dispose of used menstrual management materials” [5].

Across the globe, researchers acknowledge that many girls and women in low- and middle-income countries face various barriers to managing menstruation [6-9]. They include but are not limited to irregular menstrual patterns, lack of proper sanitation facilities including girl-sensitive toilets, water, and soap; unhealthy cultural practices that segregate menstruating girls, teasing and ridicule from peers, and lack of access to analgesics for pain relief. These affect their health, their potential freedom to access education, employment, overall safety and quality of life [10]. Globally, about 52% of the female population is of reproductive age [5]. This implies that at least, half of the global population menstruate as part of their life cycle and there is a wide range of experiences according to each individual peculiarity, bio-physiological, socio-cultural, and psychological.

Evidence suggests that pubescent girls are generally unaware of the changes to expect during menstruation. Dwivedi *et al*. reported that 68.1% of adolescents in Indonesia are unaware of menstrual matters [11]. A low-level of knowledge regarding reproductive health inflicts different kinds of problems, including confusion when dealing with changes occurring in the body, inability to understand the menstruation cycle, and disorders of menstruation. This in turn may lead to school absenteeism, poor knowledge regarding reproductive hygiene, alienation in society associated with false myths, and depression [12,13], as well as wrong diagnostic and management approaches of adolescent menstrual problems.

Insufficient attention has been paid to how menstruation affects girls at school in Bangladesh and the challenges it presents to their school attendance, academic performance and right to education. Inadequate MHM practices contribute to 40% of school absences among menstruating girls in Bangladesh and menstruation is still regarded as something unclean or dirty since the issue of menstruation is rarely mentioned publicly, due to cultural taboos [14]. Other countries like Pakistan [15], Indonesia [1], Ghana [9], and Guinea [16] shared evidence of poor menstrual facilities, menstrual-associated depression, poor disposal facilities, and menstrual taboos respectively, resulting in school absenteeism.

A study in Nigeria with 494 menstruating school girls reported that menstruation usually puts tension on 46.2%, disrupted work at school in 38.9%, at home at 42.9%, and absence from school for at least one (1) day in the last six (6) months among 15.6% of respondents [17]. The study further suggests that school-age girls lack access to water, hygiene, and sanitation (WASH) facilities, are insufficiently informed about reproductive health, the process of menstruation, the physical and psychological changes associated with puberty and coming of age. UNICEF opined that menstruation is generally perceived by most people, especially in rural Nigeria as unclean, filthy, dirty and shameful and these perceptions cloud information on mensuration with myths, secrecy, and embarrassment [18]. Across the continent, associated myths include that men must never sleep in the same room nor eat meals prepared by a menstruating wife. Others include that washing and disposing of used pads/rags could expose women to witchcraft.

Studies posit that lack of WASH in schools contributes adversely to the health and educational development of school children. Shah *et al*. reported that 40% of menstruating students come late to school, absenteeism from school 40%, poor performance in examinations 42%, low academic performance, 44% [13].

Menstrual health and hygiene (MHH) encompass both menstrual hygiene management and the broader systemic factors that link menstruation with health, well-being, gender equality, education, equity, empowerment, and rights. Menstrual health and hygiene are therefore important contributions toward achieving sustainable development goals, the fulfillment of girls´ and women´s rights, and a key objective of the Sustainable Development Goals (SDGs) [19]. SDG 6.2 acknowledges the right to MHH with the explicit aim to, “by 2030, achieve access to adequate and equitable sanitation and hygiene for all and end open defecation, paying special attention to the needs of women and girls and those in vulnerable situations”. Other goals associated with menstrual health and hygiene include SDGs 3, 4, 5, 8, and 12 [20]. It is therefore obvious that, without considering the need for safe and dignified menstruation, the achievement of quality education (SDG 4), gender equality (SDG 5), clean water and sanitation (SDG 6), decent work and economic growth (SDG 8) and responsible consumption and production (SDG 12) will remain a mirage.

Around the world, a growing coalition of academics, donors, non-governmental organizations (NGOs), United Nations agencies, grassroots women´s organizations, multinational feminine hygiene companies, and social entrepreneurs are mobilizing to bring attention and resources to address menstruation and menstrual hygiene management (M&MHM) among girls and women in low and middle-income countries (LMICs) [21-23]. These and other global bodies have been advocating for improved provision of puberty guidance, sanitary materials, water, and sanitation facilities for girls and women across the globe especially in LMICs with limited results. Previous researchers mostly medicalized menstruation or studied dysmenorrhea and other menstrual disorder. Others explored experiences with menstrual hygiene management. The paucity of studies that explored the experiences of pubescent girls with menstruation-associated shame, embarrassment, stigma, and absenteeism (SESA) and its probable contribution to school dropout informed this study. Exploring pubescent´s experiences with menstruation-associated SESA provided first-hand information, aided understanding of the meaning of menstruation to pubescents, and promoted understanding on issues related to menstrual-associated shame, embarrassment, and absenteeism.

Summarily, this study proposed a model that serves as a care pathway for parents, teachers, and caregivers of pubescent girls and health care practitioners in managing menstrual issues that will meet the age-sensitive needs of the school-going adolescent in a fast-changing world.

## Methods

**Research design:** the design for this study was the constructivist grounded theory. Grounded theory (GT) is a flexible qualitative research method that is characterized by systematic, inductive, and iterative approach for gathering and analyzing data [24]. GT design is based on symbolic interaction theory and explores how people define realities and how their beliefs are related to their actions [25]. In GT, the reality is understood by attaching meaning to situations or events that bring meaning to life processes and events. A large body of research on menstruation adopted a positivist paradigm which seeks to understand phenomena in quantitative terms such as how variables interact, shape events and cause outcomes. Viewing menstruation from such a paradigm fails to appreciate factors influencing how menstruation is experienced and understood in different contexts. Therefore, there was a need for qualitative data that allowed for an exploration of how menstruation associated shame, embarrassment, stigma and absenteeism is understood and negotiated within the school system by female pubescents. By using a qualitative design and interpretivist paradigm, this study focused on the complex way in which pubescent students attach social meaning to their experiences of menstruation and how a wider set of interacting contextual factors influence their experiences of menstruation. The qualitative design was selected particularly because it is context-sensitive and is in line with the social constructionist and socio-ecological approaches towards understanding menstruation behaviour from the participants´ view.

The researcher followed the philosophical view of relativist ontology, constructivist epistemology, symbolic interactionism, and pragmatism. Methodological sensitivity was also ascertained by reflecting on the significance of the research, background, aim of the study, the intent and direction of the research, context, and findings from the introductory literature search. The STROBE checklist; Consolidated Criteria for Reporting Qualitative Research (COREC) was used in reporting.

**Research team/reflexivity:** the principal researcher served as the moderator during the focused group discussions, and two experienced, female interviewers fluent in English and Igbo languages who received training, including a pilot interview in a nearby school, assisted the researchers in the focused group discussions and all interviews. The principal researcher is a Ph.D. student, a qualitative researcher, who had developed a mid-range theory using grounded theory methods. The researcher had no prior relationship with the students, however introducing herself as a nurse, an academic and a woman who has experienced menstruation for some decades gave the students some confidence to express themselves freely. On the other part, the researchers did not impose personal experiences on participants but rather used that to explore further into details that pubescents had reservations about without hurting their pride.

**Recruitment of participants:** participant´s recruitment was purposive, 20 pubescent girls aged 13-18 from semi-urban and rural co-educational schools participated in the study. Participant must have menstruated for at least one year to be eligible to participate, must be fluent in spoken English and Igbo languages. They must also have consented to participate in the study and be available for all interviews. Other specific criteria for judging eligibility included: 1) participant must have experienced a leak/stain in school; 2) must have been absent from school for at least 4 days in the past 6 months for menstruation-related issues; 3) had experienced teasing, ridicule, or shame and or have been stigmatized as a result of menses. Participants must meet any two of the above three criteria in addition to the general criteria to be eligible.

**Sampling technique:** purposive sampling technique and snowballing were used. Participants were initially purposively sampled using a matrix to achieve variability across age groups. One hundred and twenty participants were initially recruited based on age, school, and class, a questionnaire on menstrual characteristics was administered, and this was used to ascertain if participants met inclusion criteria. Ninety-five (95) were dropped for not meeting the inclusion criteria. Therefore, twenty-five (25) girls in school who met inclusion criteria were recruited, and using snowballing, an additional five (5) girls who dropped out of school for menstruation-associated issues were also recruited. Ten were eventually dropped for non-completion of all interviews, poor expression, and responses. In all 20 participants informed the study.

**Instruments for data collection:** data was collected using focused group discussions (FGD), in-depth interviews (IDIs), key informant interviews (KII), and observations. Three (3) focused group discussions were held. Each focused group interview had a minimum of 6 and a maximum of 8 participants. The instruments were designed in two parts. Part 1 contained six (6) researcher-structured items on the socio-demographics of participants and nine (9) items on menstrual characteristics which provided a basis for judging if participants met the inclusion criteria while part two contained twelve (12) semi-structured questions used as an interview guide in exploring experiences with menstrual associated shame, embarrassment, stigma, and absenteeism.

**Methods of data collection:** the interviews were conducted face-to-face in English and Igbo languages depending on the participants´ preferences. The principal researcher and two assistants conducted the interviews and all interviews were captured on tape. Participants were approached and screened for eligibility and consent was obtained from the school principals, parents, and the participants.

Interviews were conducted within the school, when classes were not going on, such as during break periods, during sporting activities, and at participant´s preferred places, away from noise and distractions. In-depth interviews (IDIs) explored the participant´s bio-physiological functioning, associated psycho-social issues, and experiences of menstrual pain and symptoms, followed by the experiences with managing menses at school; and thereafter, events causing embarrassment, shame, stigma and absenteeism were explored. Prompts, paraphrasing, and nods were used to stimulate communication, and theoretical memos were written. The interviewer used the zig-zag method of data collection where the interviewers kept going back and forth until there was evidence that no new themes were arising meaning that there is data saturation. Data collection and analysis occurred concurrently until data saturation. Interviews were conducted at least twice for each participant. A total of 52 interviews were held with each lasting about 40 minutes. The process of data collection, simultaneous data analysis, and coding provided an analytic lens early into the study and directed on what data to collect next.

**Ethics approval:** ethical approval for the conduct of this study was obtained from the University of Nigeria Ethics Committee following strictly the guidelines by the Senate Research Committee (NHREC/05/01/2008B-FWA0000245 8-1RB00002323). Informed consent from all of the participants, schools and parents were obtained prior to data collection. Participants were fully informed about the purpose of the study, their roles in the study and thereafter, required to sign a consent form stating that they understood the information provided to them about the study. Participants were made aware that participation is completely voluntary and they could leave at any point during the study if they so wish, with no negative consequences for doing so. Anonymity and confidentiality of their information were ensured; as there was no identifying information linked to participants rather pseudonyms and codes were used. Permission for the use of a digital recorder was obtained from participants.

### Data analysis

**Transcription of data:** interview data were transcribed verbatim from the tape into a hundred and three (103) paged document. Information about tone, expression, and pauses were included in transcripts, as well as gestures and other descriptions by participants. Transcribed data were read line by line severally to develop conceptual codes; codes were grouped to generate categories. Thematic analysis informed by the grounded theory design was used to identify emerging themes/categories and their properties. Data were analysed manually using a conceptual file by developing a physical file for each theme/category. According to Chamaz, initial sampling in grounded theory can only serve to get you started, whereas theoretical sampling is the compass that directs the researcher in theoretical elaboration and refinement and a grounded theory emerges after going through at least two phases of coding; initial and focused [23].

**Coding of data:** axial to open coding paradigm was used as a framework. The researcher, and a co-coder coded the over a 100 paged transcript. Coding was done in two phases initial and focused. Initial coding was used to identify data to be collected next, refine the data and further direct the researcher. Key issues, events, and activities that could serve as categories or properties of a category were identified and coded, and each code represented context, conditions, actions, interactions, and outcomes [26]. Focused coding was used to separate, classify and synthesize large data. This ensured data understanding, development of categories and subcategories, and the emergence of the core or central phenomenon.

These codes were subjected to code-to-code constant comparison analysis, code with emerging category comparison analysis, and category with category constant comparison analysis. During the entire data collection, theoretical memos were written to capture participants´ nonverbal cues, theoretical questions, and coding summaries and to formulate hypotheses. These were used to monitor and stimulate more codes, and as a basis for theory integration and ultimately theory generation [27].

## Results

**Description of participants:** participants were aged between 8 and 20 years old with almost two third 12(60%) in the14-16 age range. Majority 16(80%) were in senior secondary classes, 12(60%) lived with parents, 14(70%) lived in semi urban residence. Nineteen (95%) were single. Mothers´ educational qualification was mostly secondary school with 10(50%) of the population. 16(80%) of the population have fathers with a secondary level of education and above (Table 1).

**Table 1:** socio-demographic characteristics of pubescents recruited from two selected semi-urban and rural co-educational secondary schools in Enugu State, (Nigeria) from January 2022-March 2022 (N=20)

Variable	Frequency	Percentage (%)
**Age**		
8-10	1	5
11-13	4	20
14-16	12	60
17 and above	3	15
**Class**		
JSS 1-3	4	20
SS 1-3	16	80
**Student lives with**		
Parents	12	60
Relatives	6	30
Others	2	10
Place of residence		
Semi-urban	14	70
Rural	6	30
**Marital status**		
Single	19	95
Married	1	5
**Mother’s educational status**		
No education	2	10
Primary education	6	30
Secondary education	10	50
Tertiary education	2	10
**Fathers educational status**		
No education	1	5
Primary education	3	15
Secondary education	13	65
Tertiary education	3	15

JSS: junior secondary school; SS: senior secondary

**Menstrual characteristics of participants:** thirteen (65%) of participants attained menarche between 11-13 years old. Most of them 13(65%) reported having a flow of 4-5 days´ duration, 13(65%) were unaware of their length of cycle, and 11(55%) experienced dysmenorrhea. A majority 18(80%) had information prior to menarche, and 12(60%) reported that they had no support at school during menstruation. All 20(100%) agreed to having experienced a leak or stain in school, 19(95%) have felt embarrassed and ashamed as a result of menstruation, 19(95%) reported that they have missed school within the past six (6) months as a result of menstruation (Table 2).

**Table 2:** menstrual characteristics of pubescent girls recruited from two selected semi-urban and rural co-educational secondary schools in Enugu State, (Nigeria) from January 2022-March 2022 (N=20)

Variable	Frequency	Percentage (%)
**Age at menarche**		
8-10 years	5	25
11-13 years	13	65
14-16 years	2	10
>16 years	0	0
**Duration of flow**		
1-3 days	2	10
4-5 days	13	65
>5 days	1	5
**Length of cycle**		
<28 days	2	10
28 days	1	5
>28 days	4	20
Do not know	13	65
**History of associated pain (dysmenorrhea)**		
Yes	11	55
No	8	40
Not sure	1	5
**Any information prior Menarche**		
Yes	18	80
No	1	5
Not quite	1	5
**Support at school during menstruation**		
School	0	0
Teacher	2	10
Students/friends	4	20
Others	2	10
No support	12	60
**Experiences with leak/stain**		
Have experienced leak/stain yes	20	100
No	0	0
**Have felt embarrassed/shame with menses**		
Yes	19	95
No	1	5
**Missed school during menses**		
Yes	19	95
No	1	5

**What are pubescents’ experiences with menstruation and menstrual hygiene management at school?** Perception about menstruation both positive and negative among girls experiencing menstruation was explored and finding showed that it is usually constructed based on biology, exposure, experience, cultural connotation, and practices. This could influence the bio-functional, social, and psychological response to menstruation and menstrual hygiene management. Exploration of pubescent´s experiences with menstruation and menstrual hygiene management exposed that there are a few who feel that menstruation cannot be managed in school. We interacted with two school drop-outs who held that view and they expressed thus: *“The first day I saw” it”, I was scared, luckily I was at home, then when I saw” it” the day I was going to school, I was a different person, I was feeling scared, afraid that “the thing” will leak out, afraid that teachers will ask me to stand up in class or give me a duty to perform, then I leaked in class and the boys saw “it” and teased me, afterwards, I started missing school on my bleeding days then I decided to stop...”* (P: 7). For participant 19, she stated: *“...My mother usually says that during her “time”, immediately she started menstruating, she was given out in marriage. So, when I started, I looked forward to going to my husband´s house, I never went to school on the days I was menstruating, then I got pregnant at 14 years and got married”*.

Knowledge of menstrual characteristics in terms of length of cycle, duration of flow, and amount of flow was explored, however, majority of the participants were unaware of their menstrual characteristics resulting in poor menstrual management practices and behaviour. *“…I think something is wrong with me because my period usually starts at any time. My friends usually know when to expect their period but my own will always come out anytime and most times I am embarrassed…. I usually come with menstrual materials but it doesn´t come on those day, only on days that I don´t have materials´* (P: 4).

Other participants shared similar experiences. For participant 11, she stated: *“…I do not know what you mean by 28 days or how to count the days, what I know is that sometimes, I feel sick “somehow” and after sometime, “it” will start. Before, I was always staining my clothes and they were always teasing me, it affected me before and I was missing school but now I am “cool” …”*. Participants in this study are concerned that they lack the basic knowledge of how their body function. Some participants question their biological clock (circadian rhythm), for participant 13, she expressed thus: *“… my friend starts menstruating on Fridays and by Monday, she has stopped but my own will come any day from Monday to Sunday, I think I am sick…”*.

**What are pubescents´ experiences with menstrual-associated shame, embarrassment, stigma, and absenteeism?** The study explored pubescents´ experiences with menstrual-associated shame, embarrassment, stigma, and absenteeism. Participants seem to implicate rule enforcement in schools and the mode of discipline as encouraging menstrual-associated shame, embarrassment, stigma, and absenteeism. Participants relayed that school uniforms are chosen without regard to menstruation and some school activities are designed without regard to the menstrual needs of the pubescent. *“They expect us to wear white shorts and tops on Tuesdays and Thursdays in a school without toilet facilities or a changing room. How on earth do they expect us to cope? And if you wear the school uniforms on sports days, you will be “doomed” I think they are just being unfair”* (p: 8). The participants expressed that while respect for constituted rules/authorities should be paramount in school that there is a need for the understanding of the social, psychological, and social needs of the menstruating pubescent. Basic rules like standing up while answering questions, do not leave the class before the end of class among others should be enforced with some reservations, care, and concern in order to achieve desired objectives.

A participant shared her experience, she stated: *“… I was assigned a seat in the front row, but usually during my period, I usually switch sitting position with the girls at the back because I usually want to check my back whenever I stand up. Some teachers will always insist you sit in the front row against your wish, making you uncomfortable. So, to avoid their embarrassment, I usually stay out of school during my menstruation”* (P: 3). The data implied that some school authority while discharging their roles encourage absenteeism from school. Another participant shared her experiences as thus: *“My embarrassing moment came when we were having classes, our teacher asked me a question, but since I was not feeling too confident in myself, I answered the question sitting down but she picked offence. She insisted that I will stand in front of the entire class. I resisted at first, but when she insisted further, I stood up and tears rolled down my eyes as a girl came to cover me with her sweater. I felt pain, embarrassed, ashamed and victimized…”* (P: 2).

Forced participation and compliance with rules were also evident in the data. Participants expressed dissatisfaction with forced demand for participation and compliance. A participant shared an experience from her previous school: *“… The most embarrassing day of my life was the day our games master [male teacher in charge of sports] insisted that I participate in a race, it was my second day and I was also feeling a little sick. Four (4) of us who were sitting at a corner were forced to participate while other students watched and cajoled us. A few minutes after the race, I watched helplessly as blood rushed down my legs, I believe everybody saw it, after that incident, I never touched my feet at that school again…”* (P: 9). Other students expressed displeasure with the teacher/student interaction which they believe should be cordial, informative and enlightening.

**What were the bio-psycho-social influences on pubescents´ experiences with menstrual-associated shame, embarrassment, stigma, and dropout?** Participants have varied experiences on menstrual hygiene management influencers. They include but not limited to scheduling of school activities including classroom teaching and examinations, sporting activities, availability/unavailability of menstrual support facilities and relationship with prefects and teachers. For classroom activities, classes last 40 minutes, there is 3-4 classes before break time and varied experiences are shared. Examinations are also usually planned to the satisfaction of the students. *“…For me, I do not have problem with the class schedule in senior classes, sometimes you don´t have classes in the morning and you can afford to join later classes”* (P: 5).

Examination schedules according to participants are usually flexible and may not interfere with the menstrual management of the pubescent. *“We usually have 2-3 papers a day and there are breaks in between such that one can attend to her menstrual need, however, we usually see girls get stained after exams but I think that has to do with poor management”* (P: 8). However, some participants shared their experiences: *“I usually feel that my flow is very heavy during the examination and I usually get stained. I remember staining my clothes at least thrice in the past three years during examination and in one of the occasions (the first), I didn´t come with a sweater to cover my back (P: 11)”*.

Participants expressed that lack of WASH facilities in school affect their menstrual management especially during examination. Majority expressed that they do not like to change their menstrual absorbent materials in school due to lack of facilities so they are forced to use thick materials as menstrual absorbent to avoid stains. These materials are used for the whole day at school. Majority, also do not perceive the odour from their menstrual absorbent materials until when they are teased or confronted. Some key informants shared their experiences with girls in the classroom. A teacher and a male student expressed that girls´ smell in class, making other students uncomfortable. This they said result in teasing and or cracking jokes that is aimed to correct the student without mentioning name. *“When you sit near some girls, they smell, most times we cannot tell them that they are smelling, we may walk away from them, or change sitting position from them or sometimes especially during examinations, we sit and endure”* (boy 17, classmate). A participant corroborated this assertion. *“In my junior secondary three (JS 3) class, I sat in between two boys in the class, the boy sitting by my right side left our seat and was discussing with the boy in the front row, I perceived that they were talking about me because they were stealing glances at me while talking in low tones. Then the other boy starting shouting in class, “we will soon start bathing girls who smell in school here in the classroom”, I felt uneasy but sat calmly until another boy mentioned my name. Some girls came to my rescue and the situation became messier. I left school that day and did not come to school for about 2 weeks. Till today, they still tease me”* (P: 15).

Participants expressed that activities in school are usually monotonous and does not give room for choices leaving the menstruating students with the options of falling out with authorities, bolting away from school or facing the consequences of engagement especially during sports and manual labour.

**Researchers´ observations:** during the process of data collection, the researchers observed as students were being chased out from the classroom into the school field by the male sport teacher and the games prefects. The team observed that some girls reluctantly walked into the school field and reluctantly stood or sat under the neem tree. At that moment certain questions raced through the researcher´s mind which was shared with participants in further interviews: 1) what other extra-curricular activities go on in the school during sport? 2) What are the available options for students who may not be disposed to take part in the sporting activities? 3) How do menstruating students cope with sporting activities in schools? Recurrent response among participants in subsequent interviews indicated that there is non-availability of any other activity at the time of sport, everyone is therefore expected to be in the school sport arena. There is no music class anywhere, no debating club and the sports options are limited to match past, long jumps, short and long distant races and of course football. This puts a menstruating school girl at a disadvantaged position and may force her to stay out of school on such days or risk falling into the bad book of teachers.

**How can the pubescent´s menstrual experiences be enhanced?** Menstrual support through provision of menstrual enablers is essential for navigating through menstrual challenges. Participants expressed that there is need for support in terms of provision of menstrual materials, facilities for menstrual hygiene, menstrual information, care and concern. *“We most times cannot afford disposable pads and safe underwear. Sometimes mothers will tell you to use old wrappers, if we can get the support, I think we will have a good menstrual experience”* (P: 4). *“It is important for the school to build toilet facilities and encourage teachers to teach the students how to manage their menstruation and not teasing the students or using our menstrual challenges to crack jokes”* (P: 8). *“For me, I think some teachers are not supposed to be in this school, they are sometimes the ones sending students away from school by the way they treat students during menstruation”* (P: 17). *“My major challenge during menstruation is changing behind the classroom and buying water to wash hands after changing, if we could have support from parents, teachers and the government, I think we will have good menstrual experiences and students will not be staying out of school during menses”*.

**What is the meaning of the experiences with menstrual associated shame, embarrassment, stigma and absenteeism?** Four (4) categories and eleven (11) sub category emerged from the data. These formed four themes and explained the bio-physiological, socio-cultural and psychological processes associated with menstrual shame, embarrassment, stigma, absenteeism and school drop-out (Table 3).

**Table 3:** emerging categories, themes and sub-themes from the data on the experiences with menstrual associated shame, embarrassment, stigma and absenteeism among pubescents’ in a rural and semi-urban co-educational secondary schools in Enugu State, Nigeria

s/n	Categories	Themes	Sub-themes
1.	Bio-physiological functioning	Individuals’ bio-physiological status, knowledge of menstrual health and menstrual characteristics, influence pubescent menstruation behavior	1) Bio-physiological status; 2) knowledge of menstrual characteristics; 3) menstrual health status
2.	Regimenting school	Regimenting school through strict rule enforcement, punishment/motivation, forced participation and compliance have influence on the menstruation behaviour of menstruating pubescent in the school	1) Rule enforcement; 2) punishment/motivation; 3) forced participation; 4) forced compliance
3.	Regulating activities	Regulating school activities including academic activities/examinations, sporting and other extra curricula activities with/without options may influence the behavior of a menstruating pubescent in school	1) Classroom activities/exams; 2) sporting/other activities
4.	Providing menstruation support	Providing menstrual support by individual and institutional efforts to menstruating pubescent may influence menstruation behavior	1) Individual support; 2) institutional support

These themes are generally denoted by the term “menstrual influencers and have direct interactions with menstrual enablers and the institutions”. Theme 1: bio-physiologic functioning including individuals´ biological make-up and functioning, knowledge of menstrual health and menstrual characteristics, influence pubescent´s menstrual behaviour. Theme 2: regimenting school by strictly enforcing rule, punishments, forced participation and forced compliance have profound influence on the menstrual behaviour of the pubescent girl at school. Theme 3: scheduling school activities including classroom teaching/ exams, sporting and other extra curricula activities with or without option/choices may influence the behaviour of a menstruating pubescent in school. Theme 4: menstrual support through provision of menstrual enablers is essential for navigating through menstrual challenges.

**The core category:** menstrual support is the category that constantly emerged in all the theme because for every menstrual influencer (theme), individual and institutional support are needed to navigate through the menstrual threat towards a positive or negative menstrual behaviour. Support system needed by pubescents include adequate menstrual informational support, menstrual absorbent material support, provision of facilities that support dignified menstrual experiences (WASH), care and concern for the pubescent´s physical, social and psychological well-being. These menstrual supports are denoted by the term ‘menstrual enablers’. Exploration of the experiences with menstrual associated shame, embarrassment, stigma and absenteeism among pubescents were grounded in the menstruation behaviour influencer model.

## Discussion

**Figure 1:**
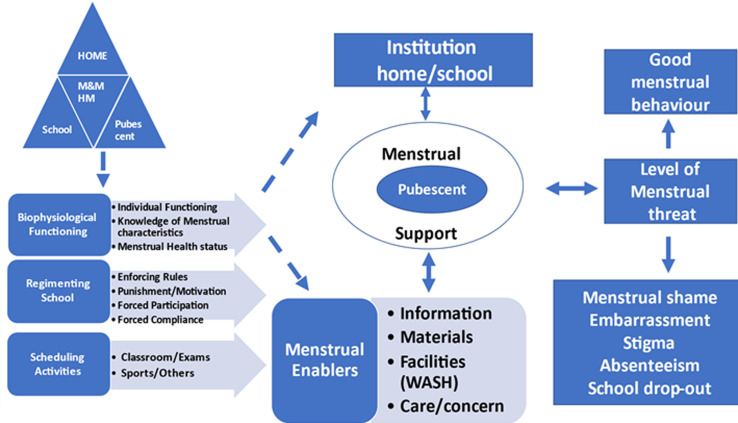
the menstruation behaviour influencer model: a grounded theory of the experiences with shame, embarrassment, stigma and absenteeism among pubescents in rural/semi urban co-educational secondary schools in Enugu State, Nigeria

**Experiences with menstrual associated shame, embarrassment stigma and absenteeism:** the exploration of school going pubescents´ menstrual experiences yielded rich data that culminated into the model of menstrual behaviour influencer with four (4) themes and eleven (11) sub themes. Menstrual support was the core theme around which most of the other themes revolved. Succinctly put, “if menstrual support is available to menstruating pubescent girls, there will be an overall good menstrual behaviour, but when it is lacking, poor menstrual behaviour resulting in shame, embarrassment, stigmatization, absenteeism and school drop-out may occur”. Based on the above, the menstrual influencer model was proposed (Figure 1).

**Description of the model:** structurally, arrows were used to describe the directions and mode of interactions within the model. Broken arrows were used in the model to indicate the direction of flow of energy within the model, two directional arrows were used to highlight the interactions between variables and thick one directional arrow is used to indicate the consequences of the interactions. The structure of the model depicts that there is a direct relationship between the identified themes (menstrual influencers) and the central phenomenon (menstrual support), also between the menstrual enablers and the central phenomenon. Menstrual support has a two-way interaction with the institutions and menstrual enablers. The consequences of the interaction are seen indicated by the thick single arrow.

Theoretically, the model starts with the interactions in the home-pubescent-school triad on menstruation and menstrual hygiene management. The model is described in three domains, the bio-physiologic, the socio-cultural, and the psychological domains. The bio-physiologic domain considers the biologic and the physiological functioning of the pubescent, the parents and the teacher and the influences on menstruation and menstrual hygiene management of the pubescent. The socio-cultural domain considers the norms in the institutions (both home and school) and the influences on pubescent´s menstrual behaviour. The psychological domain X-rays the consequences of the interaction between the bio-physiologic domain and the socio-cultural domain on the menstruating pubescent´s psychological health. These align with the biopsychosocial model.

It was evident in the data that individual´s biological make-up, exposure, and sociocultural context have influence on pubescent menstrual behavior. In support, Tanton *et al*. reported an association between menstruation and feeling of anxiety especially among girls with poor knowledge of the biology of menstruation [28]. The study identified that menstrual anxiety resulting from poor knowledge of menstruation, sociocultural indoctrinations, and menstruation-associated myths influence the pubescent´s menstrual behaviour. This also agrees with Tanton *et al*. which stated that menstrual anxiety was strongly associated with various aspects of the socio-cultural context, particularly negative behavioural expectations, lack of menstrual confidence, and shame (anxiety about being teased), possibly reflecting an internalised stigma [28].

Furthermore, poor information on menstruation affects the menstrual behavior of the pubescent girl. In support of the above, Chandra-mouli *et al*. assert that Menstrual misinformation from the main sources (mothers), who lack sufficient and accurate knowledge of menstruation and the biological process involved, pass on taboos and restrictions to adolescents, resulting in misconceptions and misunderstanding about menstruation [29]. Hennegan *et al*. further substantiated this claim, it stated, that “the socio-cultural context has a great influence on menstrual experiences, manifesting in strict behavioural expectations to conceal menstruation and limiting the provision of menstrual materials" [30]. Such are the experiences among pubescents in this study.

Puberty is a key stage in the transition from childhood and can be a challenging and confusing time, however knowing what to expect, why such changes occur, and how to navigate the changes is very important for optimal wellbeing. Menstruation through a normal physiological process can impact on the physical, social and psychosocial behaviour of the girl child and on her day-to-day activities. Feelings of maturity, being grown up, pride, excitement, worry, anxiety, moodiness, embarrassment, physical and social isolation etc. have been reported among girls at menarche and following monthly menstruations [31,32]. These experiences were expressed by students.

Participants in this study were also concerned that they lacked the basic knowledge of how their body function, and or why they experience menstrual irregularities. Menstrual cycle irregularities and primary dysmenorrhea are among the most common female complaints. Itani *et al*. opined that majority of the adolescent whose menarche is less than two years will most likely experience irregular menstruation [33]. Findlay *et al*. proposed the need for menstrual cycle profiling among staff and clinicians that address menstrual biology, especially among sporting teens, and the need for monitoring and developing awareness, openness, and understanding of the menstrual cycle [34]. Belayneh *et al*. in support of the assertion stated that poor knowledge and understanding of menstruation may lead to unsafe hygienic practice, risk of reproductive and genito-urinary tract infections, cervical cancer, school drop-out, poor academic performance and overall poor quality of life [35].

This finding aligns with the biopsychosocial model which discusses the influences of biology in terms of genetics, physiology, anatomy, epidemiology, and nutrition on the body. Participant´s bio-functional states were found to have a profound effect on menstrual perception and experiences. Participants expressed that the mode of enforcing rules in school and some teachers´ demand for respect influence their menstrual behaviours. School and classroom rules provide structures and guidelines needed to create a productive learning environment. Effective classroom management requires that the teacher enforces the rules effectively and consistently using both motivation and punishment. The purpose of punishment is to correct ill behaviors and set boundaries for acceptable behaviours. The exercise of classroom management is dependent on a lot of factors including the personality trait of the teacher, the knowledge of classroom management, the school management style and the teacher´s management style. While some teachers with permissive teaching style in class management may adopt student centered approach to teaching, autocratic teachers may demand total respect, compliance and participation from students and this has implications for girls´ retention in school.

Kirk and Sommer supported the assertion that adverse attitudes to menstruation from teachers may lead to a negative self-image among girls who are experiencing menses, and can result in a lack of self-esteem as they develop their personality as women [36]. Pubescents´ expressed displeasure with some teachers who insist that students stand to answer questions. House *et al*. stated “that some girls avoid standing up to answer teachers´ questions because of stress over leakage, smell and discomfort; or they hesitate to write on the blackboard for fear of menstrual accidents and others seeing blood on their clothes, and the subsequent shame and embarrassment this causes” [3,7].

The need for total care of the menstruating pubescent should be paramount among caregivers be it at school and other institutions. The biopsychosocial model advocates the whole person care model which refers to individual´s feelings, thoughts and behaviour as important for pubescents menstrual health. One may argue that there is need to enforce discipline among teenagers to encourage positive developmental outcome and curb truancy, however the need for positive psychosocial health cannot be neglected.

Social determinants of health as defined by WHO and the Centre for Disease Control (CDC) are the non-medical factors that affect health. The biopsychosocial model also examines how individuals live and interact in a social context, the school, family among social groups etc., whether they have financial securities, a sense of safety and how social cultural influences impact their health. School classroom interactions and extra curricula activities should reflect positive reinforcement for the leaners. A menstruating adolescent needs an environment that is safe and a healthy social system to navigate her menstrual challenges positively. Participants reported that many students do not come to school on sporting days due to lack of options for sporting and other extra-curricular activities. Others avoid school because of poor material use or poor management practices. In support of the assertion Sychareun *et al*. stated that 54 percent of 334 respondents do not have good menstrual practice [38]. Generally, girls usually do not want to engage in school sport during menstruation. 46% of young girls use period as an excuse to avoid sports at school, 39% experience a fear of period leaks or pads moving out in place, while 75% avoid sports for period shame [39].

**Navigating the challenges of menstruation and menstrual hygiene management in school among school going pubescents:** the menstrual behaviour influencer model provides a framework for managing M&MHM among pubescent in schools. This model recognizes the sensitive nature of the phenomenon, menstruation among pubescents, the silence surrounding menstruation and the socio-cultural issues that fuel shame, stigma, embarrassment and absenteeism resulting in school drop-out. Navigating the associated poor menstrual behaviour requires that the family, school and the pubescent should have a shared responsibility in maintaining dignified menstruation. While parents are saddled with the responsibility of providing menstrual information, menstrual absorbent materials, care and concern for the pubescent, the school should on the other hand provide facilities for dignified menstrual experience, factual menstrual information, emergency menstrual absorbent materials, varied experiences for students and care. There is a need for an institutionalized menstrual management pathway that guide students through menstrual management.

Students do not necessarily have to approach teachers or school prefects individually to explain their menstrual status if a functional pathway is instituted. This will remove the bottlenecks associated with maintaining hygiene in school, remove student/teacher rancour in schools, ensure dignified menstrual experiences, and keep girls in school. There could be a girl only sick bay with an adjoining handcraft room, where menstruating girls can get medications, change of material, and emergency menstrual material even if they have to pay at a later date and also engage in other extracurricular activities if sport is not an option. This would encourage discussions on menstruations among teens and slowly break the menstrual associated silence deeply engrained in cultures.

Several studies posit that menstruation is a topic clouded with secrecy, as such adequate and factual information will keep being a mirage to pubescent if the efforts being made do not address this information need in pubescent sensitive ways. Many researchers proposed providing WASH facilities, girl´s sensitive toilets and absorbent materials. My interaction with pubescent yielded rich data which implied that while provision of WASH facilities is laudable, there is a great need to provide a safe home for pubescents within schools, there is a need for a girl friendly facilities that could provide factual information on menstruation, menstrual patterns, correct use of absorbent materials, dispel associated myths and superstitions, provide medication for minor discomforts, offer emergency absorbent materials, offer facility for rest in case of moderate to severe discomfort, facility for change of absorbent material, safe method of disposal and handcrafts which serves as alternative to sports.

We propose at least a two-room girl-friendly facility with toilet facilities and safe disposal system, where the first room serves as a lying-in ward where menstruating girls experiencing moderate to severe bearable discomfort can rest a while before joining others in the classroom and a craft-room where girls can work as alternative to sporting activities without conflict with school authorities. This facility should be manned by at least a staff (a school nurse or any person with knowledge of gender issues, or adolescent health). That way, the menstrual associated shame, embarrassment, stigma, absenteeism and school drop-out will be minimized if not completely eradicated.

**Limitation of study:** this population used for the study are pubescent in semi urban and rural areas in a particular geographical region, there may be need to replicate this study across urban and rural dwellers also in other geographical areas and countries for ease of generalization.

**Recommendations:** 1) the pubescent- home-school triad is important in ensuring dignified and safe menstrual health management of the school-going pubescent, this shared responsibility should be shouldered by all concerned for positive menstrual behaviour; 2) menstrual support in terms of provision of safe absorbent materials, WASH, facilities, care, and concern should be provided to the menstruating pubescent in school; 3) girl friendly centres should be a part of every secondary school. This facility should be manned by competent hands so that girls can assess information on menarche, menstruation, reproductive health etc. and learn practical skills on the use of absorbent materials, care of used materials, access to rest facilities for moderate to severe discomfort, pain medication, emergency absorbent materials, and referral services if need be; 4) the use of the menstrual-behaviour influencer model in the assessment and management of adolescent menstrual issues is advocated for use among researchers, clinicians, parents, teachers, and the general population since it is age and gender sensitive and addresses the menstrual health needs of the adolescent.

## Conclusion

Menstrual-associated shame, embarrassment, stigma, and absenteeism are very serious issues that menstruating pubescents face in school. Parents, teachers as well as the pubescent are all implicated in this menace which robs the pubescent of her dignity, freedom of association and right to access education. However, menstrual support is needed from both the home and school to enable pubescents to navigate this all-important stage in their developmental milestone. The menstrual behaviours influencer model therefore provides adolescent sensitive care pathway that will be used by parents, teachers, clinicians, and general health practitioners in the assessment and management of menstrual-related issues among school-going pubescents.

### What is known about this topic

- *Severally studies had identified menstrual associated shame, embarrassment, stigma and absenteeism among adolescent in schools;*
- *Several agencies advocated the provision of Water, Soap and Sanitation (WASH) in schools for positive menstrual hygiene management*.

### What this study adds

- *This study proposed the menstruation behavioural influencer model that explains the menstrual influencers on the home-school-pubescent triad;*
- *The provision of WASH in school is laudable but offers limited solutions to the menstrual problem of the adolescent girl hence the need for girl-friendly facilities in school;*
- *Positive menstrual behaviour will reduce or abate the menace of menstrual-associated shame, embarrassment, stigma, absenteeism, and school drop-out*.
